# Comparison of a Rapid Light-Induced and Forced Test to Study the Oxidative Stability of White Wines

**DOI:** 10.3390/molecules27010326

**Published:** 2022-01-05

**Authors:** Emilio Celotti, Georgios Lazaridis, Jakob Figelj, Yuri Scutaru, Andrea Natolino

**Affiliations:** 1 Department of Agricultural, Food, Environmental and Animal Sciences, University of Udine, 33100 Udine, Italy; emilio.celotti@uniud.it (E.C.); lazaridis.georgios@spes.uniud.it (G.L.); figelj.jakob@spes.uniud.it (J.F.); 2 Department of Oenology and Chemistry, Technical University of Moldova, MD-2004 Chisinau, Moldova; iurie.scutaru@enl.utm.md

**Keywords:** white wine, wine oxidation, browning, light exposure, tannins

## Abstract

The oxidation processes of white wines can occur during storage and commercialization due to several factors, and these can negatively affect the color, aroma, and quality of the wine. Wineries should have faster and simpler methods that provide valuable information on oxidation stability of wines and allow fast decision-making procedures, able to trigger suitable technological interventions. Using a portable prototype instrument for light irradiations at different wavelengths and times was considered and evaluated on sensorial, spectrophotometric, and colorimetric parameters of white wines. The sensorial analysis revealed that white and light blue were the most significant, after only 1 h of irradiation. The experimental results showed that hydrogen peroxide could enhance the effect of light treatment, allowing a contemporary evaluation of the oxidation stability of wine against light and chemical stresses. As expected, a good correlation (R^2^ > 0.89) between optical density at 420 nm and b* parameter was highlighted. The synergic effect of light and H_2_O_2_ was also studied on the hydrolyzable and condensed tannins’ additions to white wine. The proposed methodology could be used to evaluate the oxidative stability of white wines, but also to evaluate the effect of some oenological adjuvants on wine stability.

## 1. Introduction

Storage and commercialization are crucial steps for both wine producers and consumers, as the wine quality needs to be ensured and maintained. White wines are usually consumed young within a year of production, to maintain their color, and fresh, fruity, and floral aroma. During storage, uncontrolled oxidation reactions can occur and cause several faults: volatile acidity increases [1], color changes from green and light yellow to brown and dark hues [2], flavor decay with many olfactory notes’ losses, and off-flavors’ formation [3,4].

Wine oxidation is a complex phenomenon since wine contains several organic and inorganic compounds, and it can be divided into enzymatic and non-enzymatic oxidation. 

The non-enzymatic process, also called chemical oxidation, has been studied in the last decades and begins with the oxidation of polyphenols containing a catechol or a galloyl group, such as catechin, epicatechin, gallocatechin, gallic acid and its esters, and caffeic acid [5]. White wines contain lower polyphenol concentrations (0.2 to 0.5 g/L), mainly hydroxycinnamic acids, but they remain crucial for oxidation-related issues in wine browning and aroma changes. The oxidation of polyphenols can lead to the formation of *o*-quinones with different degrees of polymerization and, due to their instability, further reactions can happen, and brown pigments can be formed [6]. The phenol oxidation induces, contemporary, the oxygen reduction to hydrogen peroxide, which is a potent oxidative compound that can also oxidize ethanol to acetaldehyde in the presence of transition metals [7]. Besides transition metals (i.e., Fe, Cu), other factors could affect the non-enzymatic oxidation: temperature, pH, and light exposure [8].

Light exposure can cause sensorial changes in wine, with the formation of volatile sulfur compounds and the oxidative browning spoilage. The light-induced off-flavors are mainly due to the riboflavin and are associated with the so-called light-struck taste (LST), characterized by unpleasant cabbage- and onion-like odors. The riboflavin is a highly photosensitive vitamin that induces the photooxidation of methionine generating methanethiol and dimethyl disulfide [9]. 

Light can induce the photodegradation of tartaric acid and the formation of glyoxal and glyoxylic acid. These two compounds can bin two flavanol units, forming a dimer that undergoes a dehydration and oxidation leading to a formation of yellow pigments and contributing to the oxidative browning of white wines [2,6,10]. These mechanisms of photochemical oxidation are favored by the dissolved oxygen and the transition metal ions, such as iron [9]. 

Several analytical methods have been proposed to evaluate the oxidative susceptibility or oxidation status of white wines. The cyclic voltammetry is a useful fingerprint technology for quantifying dynamic changes in wines’ composition related to their redox state [11], or investigating redox potentials of various wine compounds measuring the anodic peak intensity [12,13,14]. The oxidative mechanism can be also investigated through the detection of radical species with the so-called Electron Paramagnetic Resonance spectroscopy (EPR) [15,16]. Another approach comprises the metabolomics analysis by UHPLC/QTOF-MS systems that revealed some specific compounds able to discriminate the different oxidative statuses of white wines [17]. 

All these aforementioned analytical techniques are complex, expensive, and they require high knowledge and highly-skilled technicians. Wine quality control requires the availability of simple and rapid analytical methods, allowing regular and punctual monitoring of the different production steps and a fast decision-making procedure, able to trigger suitable technological interventions, in case of deviations from the normal winemaking conditions.

Due to the complexity of wine oxidation phenomena and the current analytical methods to evaluate the stability and shelf-life of white wines, it is necessary to have new approaches that allow more rapid, complete, and reliable evaluations. The initial aim of the present work is to evaluate, by sensorial analysis, the effect of light exposure at different wavelengths and times, using a portable prototype instrument. Moreover, the evaluation of light, chemical stresses, and their combination was carried out through spectrophotometric and colorimetric indices: optical density at 420 nm, catechins content, L*, a*, and b* parameters. The addition of hydrolyzable and grape tannins at different concentrations (50, 200, and 500 mg/L) were also considered and studied on the oxidation stability of Pinot Gris wine.

## 2. Results and Discussion

The chemical properties of Pinot Gris and Chardonnay wines are shown in Table 1. All the experimental values are in the common ranges related to the cultivation area and grape variety [18]. The oxidation stability was evaluated by the Polyphenols Oxidative Medium (POM) test, and the results revealed initial technical stability, which allow a better evaluation of chemical and light stresses affecting wine quality during storage and commercialization stages.

### 2.1. Light Exposures and Sensory Analysis

**Figure 1:**
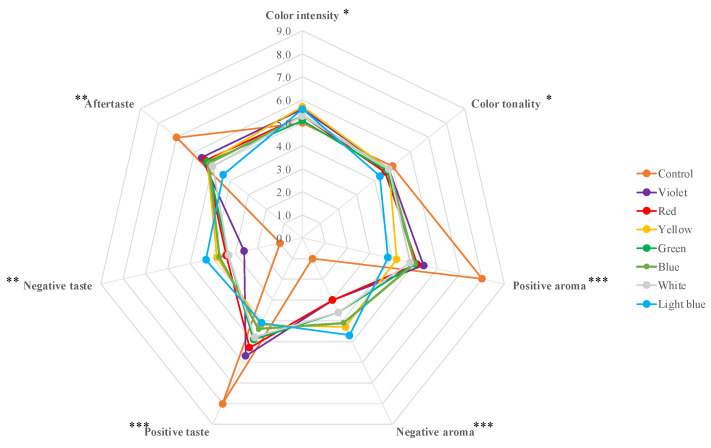
Sensorial analysis of Pinot Gris after 7 h and different light color exposures. * *p* < 0.05; ** *p* < 0.01; *** *p* < 0.001.

Figure 1 shows the results of sensorial analysis carried out on a Pinot Gris wine before (Control) and after 7 h of irradiation with different light colors: violet, blue, light blue, green, yellow, red, and white.

The radar chart reported in Figure 1 shows that the light irradiations at different wavelengths slightly affect the visual characteristics of the Pinot Gris wine. The irradiations induced a general increase of color intensity, due to an increase of amber yellow/brown color shades, and a contemporary decrease of yellow straw color tonality. The higher variation was detected after the light blue treatment with a wavelength range between 476 and 495 nm. 

The panel group of trained judges highlighted the remarkable effects of light exposures over aroma and taste perceptions. The light irradiations induced a significant decrease of positive aroma and taste perceptions, and a contemporary increase of negative sensory notes. The wine tasting after light blue treatment pointed out the highest variation on aroma, taste, and aftertaste perceptions. 

Experimental trials on Chardonnay wine pointed out that greater significant modifications on chemical composition occurred after light exposure at low wavelengths in the visible spectrum range or in the ultraviolet spectrum. Specifically, blue and violet light tonality allowed more significant changes on analytical indices, compared to red, orange and yellow light irradiation [19]. 

Considering the sensorial evaluation depicted in Figure 1, subsequently, a shorter exposure (1 h) with light blue irradiation was carried out to evaluate if shorter times are enough to induce significant effects on sensory indices.

**Figure 2:**
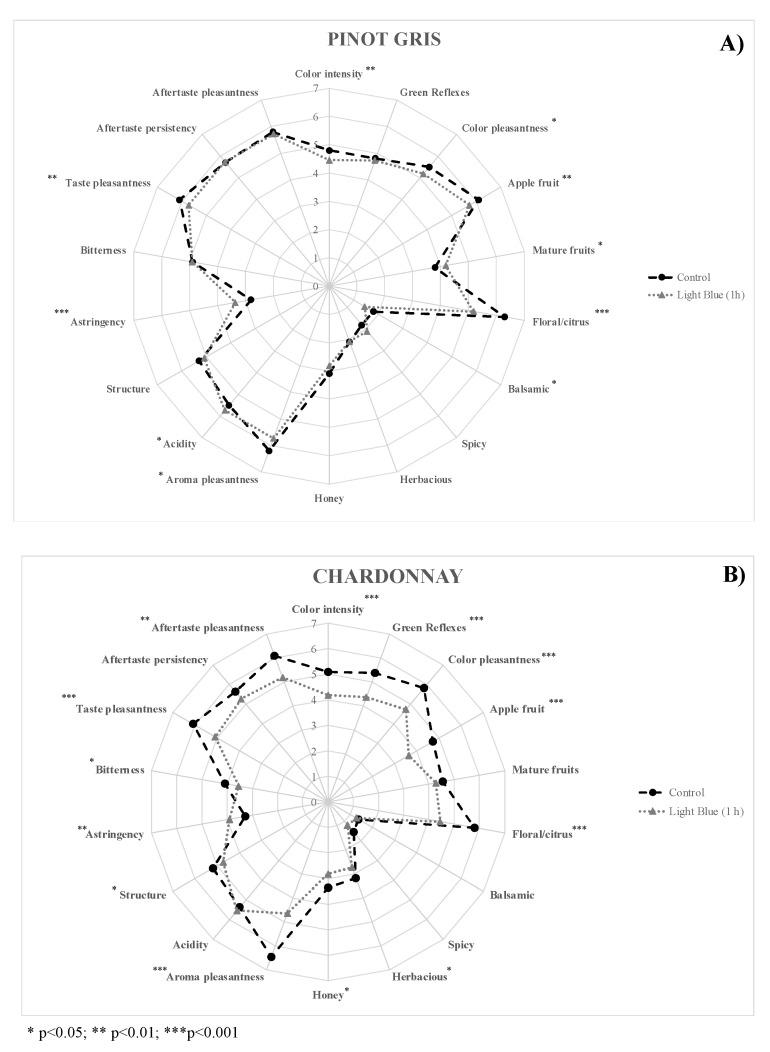
Sensorial analysis of Pinot Gris (**A**) and Chardonnay (**B**) wine before (Control) and after 1 h exposure to blue light.

A Chardonnay wine was also considered over Pinot Gris variety. After 1 h of light blue exposure, a sensorial analysis was carried out by the same trained panel group, evaluating more specific sensory descriptors on wine color, aroma, and taste. The results are reported in Figure 2.

The sensory analysis of Pinot Gris highlighted that the light blue treatment induced a common decrease of chromatic, aroma, and taste pleasantness, compared to the untreated sample. The judges evaluated a decrease of apple, citrus, and honey notes, but an increase of mature fruits and spicy perceptions. Taste evaluation pointed out a potential increase of acidity and astringency descriptors. Instead, no relevant effects were revealed about the aftertaste. Moreover, the comparison of untreated and irradiated Chardonnay wine revealed a common decrease of the considered descriptors, with a decrease of sensory quality after light blue exposure. It is remarkable, again, the significant increase of the astringency perceptions after light treatment. Polyphenols are sensitive to various physical and chemical agents, such as temperature, light, oxygen, and others [20], which can significantly affect some of their chemical and sensory properties [21]. 

The aforementioned effect is also reported for other food matrices, such as milk [22], highlighting a potential effect of light exposures on astringency perception.

**Figure 3:**
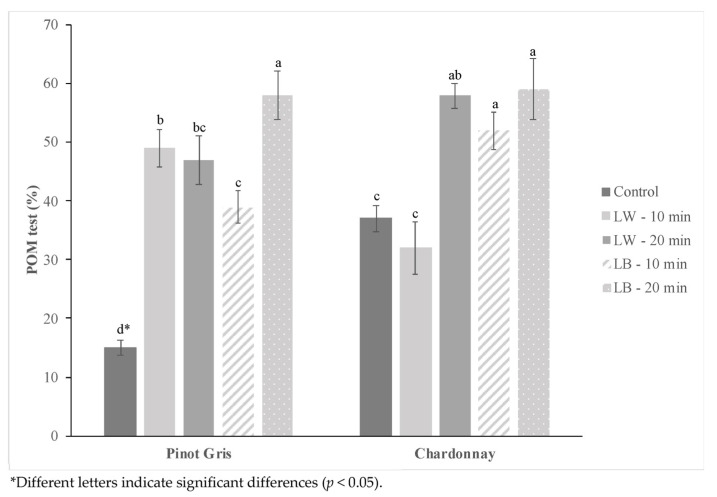
POM test value of Pinot Gris and Chardonnay wine before (Control) and after different light color (white and light blue) exposures at 10 and 20 min.

Figure 3 shows the POM test results of Pinot Gris and Chardonnay wine before and after exposure to different color lights (light blue and white) at different times (10 and 20 min). 

Shorter exposure times below 1 h were adopted to define a potential analysis protocol suitable to be directly applied in wineries, considering their conventional production times. Accelerating decision-making procedures become fundamental for wineries to evaluate the oxidation stability and the shelf-life of the wines, allowing reduced sampling time, avoiding sample storage and transport, and reducing environmental risks [23]. 

The light exposure significantly increased the oxidability of the wines, independently of wavelength and exposure time, as pointed out by experimental results (Figure 3). The light blue exposure for 20 min allowed the highest variations of POM test results: from 15% to 58% for the Pinot Gris, and from 37% to 59% for the Chardonnay wine. The increase of oxidability could be due to the photosensitivity induced by light exposures on riboflavin in the wine, a photosensitizing agent that promotes the oxidation phenomena. When riboflavin is exposed to light, it reaches the singlet state that is converted to the triplet state with an intercrossing system. Riboflavin is reduced by the acquisition of two electrons from a donor compound, that consequently undergoes oxidation [8]. 

### 2.2. Individual and Combined Stress Trials 

**Figure 4:**
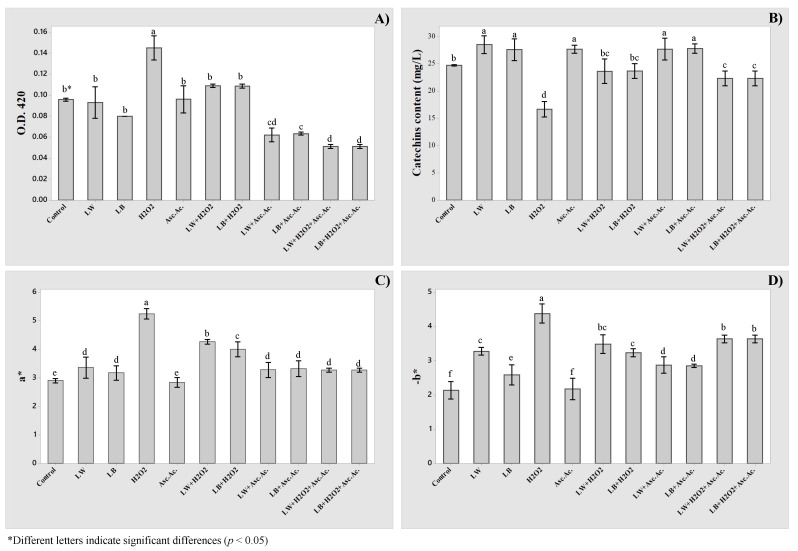
Results of OD420 (**A**), catechins (**B**), a* (**C**), and b* (**D**) before and after several chemical and light stresses.

Figure 4 shows the experimental results of optical densities at 420 nm (O.D. 420), catechins contents (C.C.), a*, and b* parameters of the Pinot Gris samples before (Control) and after different treatments: white (LW) and light blue (LB) exposures, hydrogen peroxide (H_2_O_2_), and ascorbic acid (Asc.Ac.). Moreover, also, some treatment combinations were considered to evaluate possible synergic effects: LW + H_2_O_2_, LW + H_2_O_2_, LB + H_2_O_2_, LW + Asc.Ac., LB + Asc.Ac., LW + H_2_O_2_ + Asc.Ac., and LB + H_2_O_2_ + Asc.Ac. The O.D. 420, catechins content, L*, a*, and b* parameters were adopted as response variables and used to determine the coefficient of the third-order polynomial model used for ANOVA. The estimated coefficients are given in Table 2. 

Third-order polynomial equations were found well to represent the experimental data, as indicated by the estimated coefficients of the determination R^2^ and R^2^-adj. The L* parameter was considered not suitable as response variables, considering the lowest values of R^2^ (<86%) and R^2^-adj (<80%). 

The light exposures (LW and LB) affected significantly the catechins content, a*, and b* parameter. The magnitude of regression coefficients revealed that the white light (LW) induced higher changes on analytical parameters, compared to light blue treatment (LB). Instead, the light exposures did not affect the optical densities at 420 nm, which remain the same as untreated wine (Control). 

The oxidation level of white wine in the bottle is commonly estimated by color with the extent of browning at 420 nm [8]. As reported in Figure 4, the OD at 420 nm increased significantly only after the addition of hydrogen peroxide, which is the most significant factor for all the analytical parameters (*p* < 0.001). 

The ascorbic acid is commonly employed during winemaking processes due to its antioxidant properties in a complementary role with sulfur dioxide [24]. Despite the antioxidant role, the ascorbic acid showed some pro-oxidant effects, such as its oxidative degradation to dehydroascorbic acid and hydrogen peroxide [25]. The only addition of ascorbic acid resulted statistically significant only for catechins content; no significant changes were highlighted for OD 420 nm, a*, and b* parameter. 

It is notable that the interaction between light and hydrogen peroxide addition (LW*H_2_O_2_, LB*H_2_O_2_), resulted highly significant (*p* < 0.001) for all the analytical indexes. The addition of H_2_O_2_ allowed the increase of light exposures effect, particularly when a* and b* parameters were considered. 

The hydrogen peroxide addition, also implemented in the conventional POM test, enhanced the light-induced effect on spectrophotometric and colorimetric measurements. The combination of light and H_2_O_2_ addition allowed better detection of the oxidative status of white wines and it could represent the simplest, fastest, and more complete approach than what is currently adopted by wineries.

The combination of light and acid ascorbic addition did not highlight significant changes, particularly on OD 420 nm, catechins, and a* parameter. The ascorbic acid is not significant, as reported by the significance of regression coefficients in Table 2. 

**Figure 5:**
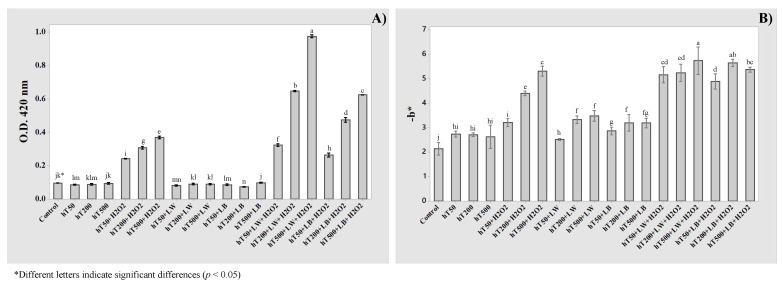
Effect of hydrolyzable tannins’ (hT) addition at different concentration (50, 200, and 500 mg/L), light exposure (LW, LB), and hydrogen peroxide addition (H_2_O_2_) on optical densities at 420 nm (**A**) and b* parameter (**B**).

As depicted in Figure 5, it is remarkable that b* parameter was the most sensitive analytical index, and it allowed the detection of single and combined stress treatments. The light exposure, specifically with white light for 20 min, increased significantly the b* parameter from 2.14 ± 0.10 (Control) to 3.28 ± 0.05 (LW). Moreover, the combination with H_2_O_2_ addition amplified the effect of white light exposure from 3.28 ± 0.05 (LW) to 3.69 ± 0.11 (LW + H_2_O_2_), and light blue exposure from 2.59 ± 0.12 (LB) to 3.24 ± 0.05 (LB + H_2_O_2_). 

Additionally, a good correlation between spectrophotometric and colorimetric measurements was found, specifically an inverse correlation between optical density at 420 nm and b* parameter (R^2^ > 0.89). 

### 2.3. Effect of Tannin Adjuvants

The use of phenols, both condensed and hydrolyzable tannins, is an acknowledged approach to limit some wine faults, such as the appearance of LST [9], thanks to their antioxidant properties as well as their ability in quenching the singlet oxygen [26,27]. During winemaking, tannins can be added on grape musts during pre-fermentative step or on finished wines as clarifying or stabilization agents. 

**Figure 6:**
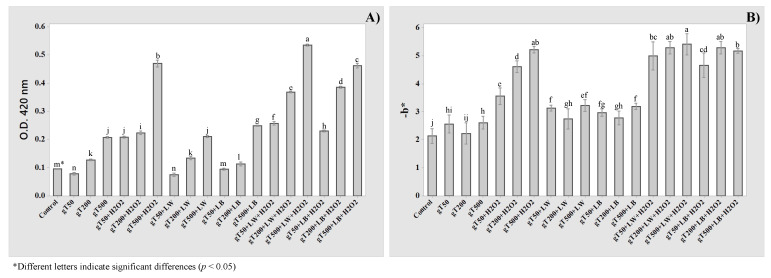
Effect of grape tannins’ (gT) addition at different concentration (50, 200, and 500 mg/L), light exposure (LW, LB), and hydrogen peroxide addition (H_2_O_2_) on optical densities at 420 nm (**A**) and b* parameter (**B**).

Different concentrations of hydrolyzable (hT) and grape tannins (gT) were added to Pinot Gris wine, and combined with light exposures (LW, LB) and hydrogen peroxide addition (H_2_O_2_). The effect of tannins’ addition and their combination with light and chemical stresses were studied on OD 420 nm (Figure 5A and Figure 6A) and b* parameters (Figure 5B and Figure 6B). As depicted in Figure 5A, the browning at 420 nm was not affected by the hT addition or by the concentration increase from 50 to 500 mg/L. An increase of b* parameter was pointed out from –2.14 ± 0.10 (Control) to 2.69 ± 0.09 (as mean value of hT50, hT200, and hT500), and no significant differences are shown due to the increase of hT concentration (Figure 5B).

Instead, the addition of grape tannins (gT) above 200 mg/L induced an increase of optical density at 420 nm from 0.096 ± 0.001 (Control) to 0.208 ± 0.001 (gT500) (Figure 6A).

As expected, the hydrogen peroxide induced a common increase of browning sample, and its effect was enhanced by the increase of tannin concentration.

It is remarkable to note the effect of light exposures (LW and LB), specifically comparing hydrolyzable (hT) and grape tannins (gT). The light exposure after hT addition did not affect the OD 420 nm, and b* parameter significantly enhanced at concentration above 200 mg/L. Instead, the light exposure showed a significant effect on both analytical parameters when grape tannins (gT) were added to Pinot Gris wine (Figure 6). As already reported in the literature [9,28], the tannins should be adequately chosen and their addition should be thoroughly evaluated in order not to alter the sensory properties of wine. Furthermore, as suggested by the experimental results, their choosing and addition should preserve or better enhance the oxidative stability of wines.

A potential synergy behavior between light exposure and hydrogen peroxide can be highlighted for both tannins’ categories, but a higher increase of analytical parameters was detected with hydrolyzable tannins (hT). Therefore, hydrolyzable tannins should be added carefully before bottling to prevent oxidation during the storage and commercialization stages.

## 3. Materials and Methods

### 3.1. Reagents and Solvents

All the solvents were of analytical grade (purity > 99%) and purchased from Sigma- Aldrich Co. (Milan, Italy). The chemical used, which include 4-(dimethylamino)-cinnamaldehyde (DAC) and (+)-catechin, were of analytical grade and purchased from Sigma-Aldrich Co. (Milan, Italy). 

### 3.2. Wine Sample

A Pinot Gris wine from Friuli Venezia Giulia region (Italy) and 2018 vintage, was used for all the experimental trials. A Chardonnay wine from the same region and vintage was also considered. Grapes of Vitis vinifera var. Pinot Gris and Chardonnay were harvested by hand at technical maturity, and were transported immediately to the winery where they were destemmed and crushed. Grape marc was immediately separated and no maceration period was carried out. An SO_2_ addition at 20 mg/L was made, and fermentation was carried out at 18 °C for 8 days and 6 days for Pinot Gris and Chardonnay, respectively. At the end of alcoholic fermentation, the wine was stored in a stainless-steel tank at 12 °C. The wine was collected in 0.75 L flint bottles, and stored in dark and fresh conditions until use. The chemical properties of Pinot Gris and Chardonnay wine are reported in Table 1.

### 3.3. Preliminary Stress Trials

**Figure 7:**
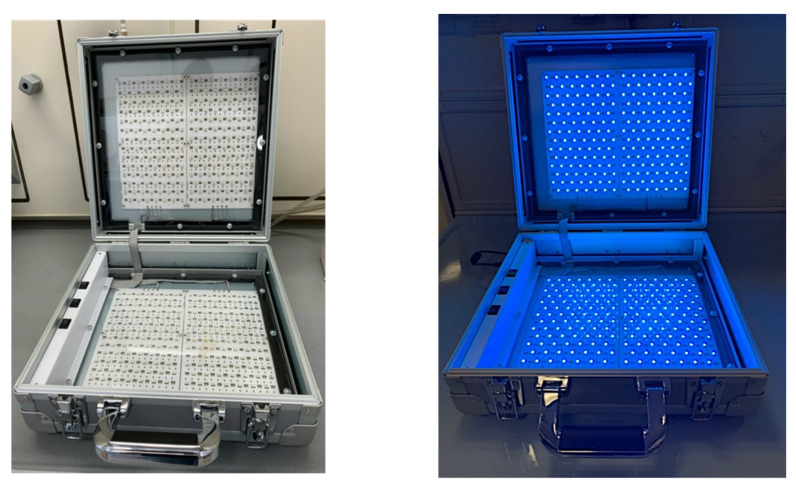
Protype instrument for light exposures.

The Pinot Gris wine was transferred in transparent glass flasks and irradiated for 4 h with 7 different color lights to evaluate the complete visible spectrum and its single ranges: violet (380–450 nm), blue (450–475 nm), light blue (476–495 nm), green (495–570 nm), yellow (570–600 nm), red (600–780 nm) and white (380–780 nm). Subsequently, the Pinot gris wine was exposed only to light blue (476–495 nm) for 1 h. A Chardonnay wine was also considered to evaluate the effect of light blue on another wine variety. All the light exposures were carried out in a portable instrument: a portable and closed case (Figure 7) constituted by multiple light-emitting diodes controlled by three switches, which allow the use of 7 different colors. The instrument was built to treat 0.75 L bottles. The case has also an air system to prevent an excessive increase of samples’ temperature due to the light exposure, and a temperature probe [29]. 

### 3.4. Individual and Combined Stress Trials

The Pinot Gris wine was stressed by different light and chemical treatments:

- Light Exposure: light blue for 20 min; (BL)
- Light Exposure: white for 20 min; (WL)
- Hydrogen peroxide: 10 mL/L of H_2_O_2_ solution (3% *v/v*); (H_2_O_2_)
- Ascorbic acid: 100 mg/L; (Asc.Ac.)
- Hydrolyzable tannins: 50, 200, and 500 mg/L; (hT)
- Condensed tannins: 50, 200, and 500 mg/L; (gT)

The tannins’ additions were carried out using commercial products: a mixture of ellagic tannins from oak as hydrolyzable tannins, and a mixture of grape skins’ and seeds’ tannins as condensed ones. Besides the single light or chemical treatment, it was some possible combinations between them were also considered.

Aliquots of 50 mL of wine samples were centrifugated at 3000 rpm for 5 min. The supernatant was transferred in 40 mL glass tubes and the chemical reagents were properly added. The light exposures were carried out in the same portable instrument previously described.

All the treatments were carried out in triplicate. 

### 3.5. Sensorial Analysis 

Pinot Gris and Chardonnay wines have been treated with different color lights, and evaluated by a sensorial analysis carried out by a panel group of 20 trained judges. The judges are researchers or oenologists experienced in wine tasting. The training of the panel group was carried out tasting wines of the same variety and category. Analysis focused on 7 general descriptors about color, aroma, and taste. Considering the results of the first evaluation, a second more detailed sensorial analysis was done considering more specific descriptors on wine color, aroma, and taste (Table 3). The judges scored the magnitude of each attribute from 1 to 9 where 1 was ‘‘low’’ and 9 was ‘‘high’’.

### 3.6. Analytical Determination

#### 3.6.1. Spectrophotometer Measurements

The Total Phenolic Indices (TPI) were calculated by measuring wine absorbance at 280 nm, according to Ribéreau-Gayon et al. [19]. The optical densities at 320 nm, related to hydroxycinnamic acid–tartaric acid esters (HCAs), and 420 nm, related to wine yellow color, were measured. All the determinations were carried out in a UV-Vis spectrophotometer (Shimadzu UV 1650, Milano, Italy), using distilled water as a control. All the measurements were performed in triplicate. 

#### 3.6.2. Flavan-3-ols’ Content

Flavan-3-ols’ content was determined according to the method proposed by Zironi et al. [30]. The chromogen reagent was prepared with 1 g of 4-(dimethylamino)-cinnamaldehyde (DAC) dissolved into 250 mL of 37% HCl and 750 mL of methanol. Next, 1 mL of diluted sample (1:25 *v/v*) was added to 5 mL of DAC solution. Then, absorbance was read at 640 nm in a UV-Vis spectrophotometer (Shimadzu UV 1650, Tokyo, Japan) against a blank prepared by substituting the sample with 1 mL of 10% ethanol solution. A calibration curve was made with several standard solutions of (+)-catechin, and measurements were carried out at 640 nm. All analyses were performed in triplicate. Results were expressed as milligrams of (+)-catechin equivalents per liter (mg/L).

#### 3.6.3. Colorimetric Measurements 

The chromatic characteristics and CIELAB parameters (L, a, b) were determined by measuring the transmittance of the wine every 10 nm over the visible spectrum (from 380 to 780 nm) using the illuminant D65 and 10° standard observer, following the official method published by International Organization of Vine and Wine [31]. All the measurements were carried out in a colorimeter Konica Minolta CR 300 (Tokyo, Japan).

#### 3.6.4. Polyphenol Oxidative Medium (POM) Test

The predisposition of wines towards browning was determined by the so-called POM-test proposed by Müller-Späth (1992), with slight modification. Briefly, 5 mL of wine was heated at 60 °C for one hour, after, 25 μL of a 3% (*v/v*) hydrogen peroxide solution was added. The browning was estimated based on the percent increase of the absorbance at 420 nm, using the following Equation (1):

(1) $$\mathrm{POM}\;\left(\%\right)=\frac{O.D.420_{H_{2}O_{2}}-\;O.D.420_{H_{2}O}}{O.D.420_{H_{2}O}}\times 100$$

where O.D.420_H_2_O_2__ is the absorbance with H_2_O_2_ addition, and O.D.420_H_2_O_ with H_2_O addition. 

All the analyses were carried out with a UV-Vis spectrophotometer (Shimadzu UV 1650, Tokyo, Japan). 

### 3.7. Statistical Analysis

All experiments and analysis were performed in triplicate and results are expressed as mean ± standard deviation. Minitab (version 17) was used for statistical analysis by one-way analysis of variance (ANOVA, with Tukey’s honest significant difference (HSD) multiple comparison test) with the level of significance set at *p* < 0.05. 

A third-order polynomial equation was used to express the response variable as a function of independent variable. The coefficients of the equation were determined by using Minitab 17 software (Minitab Inc., State College, PA, USA). The goodness of fit of the model was evaluated by the coefficients of determination R2 and R2-adj, and the analysis of variance (ANOVA, with Tukey’s HSD multiple comparison). 

## 4. Conclusions

White wine can be affected by light-dependent spoilages due to several factors, such as chemical composition, time and duration of light exposures, bottle shape, and color. Wineries can adopt several microbiological and technological approaches to prevent photochemical oxidation processes. However, the application of any oenological strategy needs analytical methods that provide valuable information on the oxidative stability of the wine.

A smart and portable prototype analytical instrument was adopted to expose a white wine at different light wavelengths. The sensorial analysis of untreated and treated samples showed significant changes after light treatments and some sensorial changes were detected, including an astringency increase. Time exposure of 20 min was considered enough to induce significant sensorial alterations. Treatments with white and light blue lights showed the highest significant changes on spectrophotometric and colorimetric determinations, specifically on optical densities at 420 nm and b* parameter. A good correlation was highlighted between OD420 nm and b* parameter (R^2^ > 0.89).

The hydrogen peroxide addition allowed an increase of light-related effects, and a synergic effect could be pointed out between these two oxidative stresses. 

The oenological strategy of tannins’ addition was also considered and evaluated on the oxidation stability of white wines. The hydrolyzable tannins are more sensitive to the combination of light and hydrogen peroxide, compared to condensed ones. The use of tannins as clarifying and stabilizing agents should be done carefully so as not to alter the sensory quality and to promote oxidation processes.

The combination of multiple stresses using a portable instrument, associated with colorimetric measurements, could represent a valuable and fast approach that can be adopted by wineries to obtain useful information on the oxidative stability of white wines. Simple and fast methodologies are needed to accelerate decision-making procedures in order to maintain and ensure the quality of the wine until the bottle opening.

## Figures and Tables

**Table 1:** Chemical properties of Pinot Gris and Chardonnay.

Chemical Parameter	Pinot Gris	Chardonnay
Alcohol (% *v/v*)	12.95	12.48
pH	3.35	3.29
Total acidity (g/L)	5.88	6.42
Free SO_2_ (mg/L)	20	27
Total SO_2_ (mg/L)	90	81
Reducing sugars (g/L)	2.16	5.74
POM test (%)	15	37

**Table 2:** Regression coefficients of the ANOVA of third-order polynomial model for OD 420 nm, catechins, L*, a* and b* parameters after light exposure (LB and LW), hydrogen peroxide, and ascorbic acid addition.

Terms	Coefficients				
	O.D. 420 nm	Catechins	L*	a*	b*
**Constant**	0.09657	24.700	18.680	2.9033	−2.1400
**Light**					
*LB*	−0.01567	2.890 ***	0.703 *	0.2700 **	−0.4533 ***
*LW*	−0.00600	3.800 ***	−0.540 *	0.4600 ***	−1.1400 ***
**H_2_O_2_**	0.04933 ***	−8.037 ***	−1.193***	2.3400 ***	−2.2400 ***
**Asc.Ac.**	0.00033	2.99	0.170	−0.0633	−0.0400
**Light·H_2_O_2_**					
*LB·H*_2_ *O*_2_	−0.0433 ***	4.124 ***	−0.480	−1.5167 ***	1.597 ***
*LW·H*_2_ *O*_2_	−0.0497 ***	3.160 ***	0.733 *	−1.4433 ***	2.033 ***
**Light·Asc.Ac.**					
*LB·Asc.Ac.*	−0.017	−2.805 ***	−1.257 ***	0.2133 *	−0.227
*LW·Asc.Ac.*	−0.028	−3.802 ***	0.267	−0.0200	0.440
**Light H_2_O_2_·Asc.Ac.**					
*LB H*_2_ *O*_2_ *·Asc.Ac.*	−0.0182	−1.554	1.723	−0.870 ***	−0.130
*LW H*_2_ *O*_2_ *·Asc.Ac.*	−0.0105	−0.503	0.230	−0.900 ***	−0.550 ***
**R^2^**	90.10%	97.98%	85.97%	98.93%	98.79%
**R^2^-adj.**	85.60%	97.06%	79.59%	98.44%	98.24%

* *p* < 0.05; ** *p* < 0.01; *** *p* < 0.001.

**Table 3:** Descriptors of sensorial analysis.

Descriptor Categories	Specific Descriptors	
	1st Sensorial Evaluation	2nd Sensorial Evaluation
COLOR	Intensity	Color intensity
	Tonality	Green reflex
		Color preference
AROMA	Positive aroma	Apple
	Negative aroma	Ripened fruit
		Citrus fruits
		Balsamic
		Spicy
		Vegetal
		Honey
		Aroma preference
TASTE	Positive taste	Acidity
	Negative taste	Structure
		Astringency
		Bitterness
		Taste preference
AFTERTASTE	Aftertaste (general)	Persistency
		Pleasantness

## Data Availability

All data has been made available through the manuscript itself.

## Sample Availability

Samples of the compounds are not available from the authors.
